# Lifetime Traumatic Brain Injury and Risk of Post-Concussive Symptoms in the Millennium Cohort Study

**DOI:** 10.1089/neu.2022.0213

**Published:** 2024-02-27

**Authors:** Kalyn C. Jannace, Lisa Pompeii, David Gimeno Ruiz de Porras, William Brett Perkison, Jose-Miguel Yamal, Daniel W. Trone, Rudolph P. Rull

**Affiliations:** ^1^ Southwest Center for Occupational and Environmental Health, UT Health School of Public Health, West Houston, Texas, USA.; ^2^ Coordinating Center for Clinical Trials, UT Health School of Public Health, Houston, Texas, USA.; ^3^ Deployment Health Research Department, Naval Health Research Center, San Diego, California, USA.; ^4^ Henry M. Jackson Foundation for the Advancement of Military Medicine, Bethesda, Maryland, USA.; ^5^ The Center for Rehabilitation Sciences Research, Uniformed Services University for the Health Sciences, Bethesda, Maryland, USA.; ^6^ Department of Pediatrics, Baylor College of Medicine, Houston, Texas, USA.

**Keywords:** acute brain injuries, brain concussion, closed head injuries, military, repeat traumatic brain injury, traumatic brain injury

## Abstract

Traumatic brain injury (TBI) is prevalent among active duty military service members, with studies reporting up to 23% experiencing at least one TBI, with 10–60% of service members reporting at least one subsequent repeat TBI. A TBI has been associated with an increased risk of cumulative effects and long-term neurobehavioral symptoms, impacting operational readiness in the short-term and overall health in the long term. The association between multiple TBI and post-concussive symptoms (PCS), however, defined as symptoms that follow a concussion or TBI, in the military has not been adequately examined. Previous studies in military populations are limited by methodological issues including small sample sizes, the use of non-probability sampling, or failure to include the total number of TBI. To overcome these limitations, we examined the association between the total lifetime number of TBI and total number of PCS among U.S. active duty military service members who participated in the Millennium Cohort Study. A secondary data analysis was conducted using the Millennium Cohort Study's 2014 survey (*n* = 28,263) responses on self-reported TBI and PCS (e.g., fatigue, restlessness, sleep disturbances, poor concentration, or memory loss). Zero-inflated negative binomial models calculated prevalence ratios (PRs) and 95% confidence intervals (CIs) for the unadjusted and adjusted associations between lifetime TBIs and PCS. A third of military participants reported experiencing one or more TBIs during their lifetime with 72% reporting at least one PCS. As the mean number of PCS increased, mean lifetime TBIs increased. The mean number of PCS by those with four or more TBI (4.63) was more than twice that of those with no lifetime TBI (2.28). One, two, three, and four or more TBI had 1.10 (95% CI: 1.06–1.15), 1.19 (95% CI: 1.14–1.25), 1.23 (95% CI: 1.17–1.30), and 1.30 times (95% CI: 1.24–1.37) higher prevalence of PCS, respectively. The prevalence of PCS was 2.4 (95% CI: 2.32–2.48) times higher in those with post-traumatic stress disorder than their counterparts. Active duty military service members with a history of TBI are more likely to have PCS than those with no history of TBI. These results suggest an elevated prevalence of PCS as the number of TBI increased. This highlights the need for robust, longitudinal studies that can establish a temporal relationship between repetitive TBI and incidence of PCS. These findings have practical relevance for designing both workplace safety prevention measures and treatment options regarding the effect on and from TBI among military personnel.

Prevalence of traumatic brain injuries (TBI) among United States active duty service members has been estimated to be between 12% and 23%,^1–3^ with approximately 449,026 of members experiencing at least one TBI between 2000 to 2021, 82% of them categorized as mild TBI (mTBI).^4^ A TBI has been associated with poor self-reported general health, more physician visits, greater missed workdays, and an increased risk of development of neurodegenerative disorders with long-term neurological dysfunction.^5–14^

Approximately 10–60% of service members report at least one subsequent repeat TBI during military service.^1,2,15^ This is problematic because TBI has been associated with an increased risk of cumulative effects and long-term neurobehavioral symptoms, impacting operational readiness in the short-term and overall health in the long term.^6,16–21^

Most individuals who have a singular mTBI, or concussion, see a resolution of symptoms from the injury within 10 days.^6,22,23^ Studies following contact sports athletes and military service members experiencing repeat TBI, however, have reported symptoms lasting an average of 4.8 years or longer after their most recent injury.^8,19,20,24–28^

While clinically diagnosed TBI records are considered more reliable than self-report, a convenience sample of 2276 individuals over two years found that 75% of respondents who sustained any TBI did not seek medical treatment, believing they did not need care.^29^ This highlights a significant limitation in relying on medical histories to complete the medical profile for a TBI patient.

When discussing military populations, further occupational complications must be considered. First, in training and combat situations, many service members may feel the need to press on as the effects of TBI, especially mTBI, are not always directly observable, which may delay or preclude seeking treatment.^30^ Second, the Armed Services are in a constant battle to overcome the stigma that follows service members reporting mental health impairment or injury.^31^ Finally, the military health system can be complicated, difficult to navigate, and service members find many barriers when chasing care.^26,30–33^

Post-concussive symptoms (PCS), defined as symptoms that follow a concussion or TBI, include physical and neurobehavioral complaints such as headache, dizziness, fatigue, sleep disturbances, problems with memory and concentration, and emotional and personality changes.^23^ The PCS differ from post-concussion syndrome, which requires a patient to experience a TBI with onset of at least three post-concussive symptoms, such as those described above, within four weeks of the injury.^23^

To date, there is no universal or gold standard method for measuring PCS after a TBI. Most military studies have measured PCS and TBI using the summed score of Cicerone and Kalmar's Neurobehavioral Symptoms Inventory (NSI)^34–38^ and the Rivermead Post-Concussion Symptoms Questionnaire (RPCQ) by King et al. ^39–43^

Existing evidence is mixed, with some studies finding no statistically significant differences between NSI score and TBI^34,36^ and others finding TBI to be positively associated with increased, or more severe, NSI scores.^6,44,45^ Findings were also mixed when using the RPCQ to measure PCS with one study finding no association found between TBI and PCS^40^ and two others that found TBI to be associated with PCS.^39,41^

Previous PCS studies in military populations are limited by methodological issues including small sample sizes, ^6,34,38,46^ the use of non-probability sampling,^6,38,47^ or failure to include the number of TBIs the worker has experienced.^6,47^ Although diagnosis of TBI has become more common with operations in Afghanistan and Iraq, few studies have considered the total count of TBI experienced by service members when examining the risk of PCS. Instead, they have defined TBI as either having it or not without consideration of the count.^1–3,16,21,28,48–51^

Some studies have collapsed the count of TBI into categories,^6,44–46,52^ but only included crude, unadjusted estimates when examining associations between TBI and PCS.^6,8,52^ Moreover, among military populations, few studies have considered military occupation as a risk factor for TBI or TBI-related outcomes, such as PCS. This is a significant limitation because existing evidence suggests TBI risk varies, not just between enlisted and officer ranks, but among different occupational categories (e.g., Infantry versus Administration).^53,54^

To overcome these limitations, we examined the association between the number of TBI and the individual and total number of PCS among U.S. active duty military service members who participated in the Millennium Cohort (MilCo) Study.

## Methods

### Study sample

Data for this cross-sectional study were obtained from the Naval Health Research Center's (NHRC) MilCo Study, an ongoing Department of Defense (DOD) study. Sampling and recruitment methods are described elsewhere.^55–59^ In brief, MilCo enrollment began in 2001, with a new round of service members recruited in panels every three years until 2011, with a total of four recruited panels.^59,60^ These analyses included participants from panels 2 (2004), 3 (2007), and 4 (2011).

To be included in the MilCo study, panel 2 required service members to have one to two years in service, panel 3 required one to three years, and panel 4 required two to five years. This study utilized data from the most recent follow-up survey (wave 4 for panel 2, wave 3 for panel 2, and wave 2 for panel 4) conducted in 2014, which was the first to assess TBI. To be included in these analyses, service members needed to be on active duty or in the Reserves/National Guard (e.g., not veterans) with complete TBI data.

Participants enrolled in the study while they were still on active duty or Reserves/National Guard. Since enrollment of each panel, some participants have retired or completed military service. There were 112,655 participants who completed the 2014 survey, including 29,286 (26.0%) participants still serving on active duty or in the Reserves/National Guard including 5100 (17.5%) from panel 2, 8565 (29.2%) from panel 3, and 15,621 (53.3%) from panel 4. There were 1023 participants missing TBI and PCS data who were excluded from analysis resulting in a final sample size of 28,263.

### Data collection

The MilCo study did not use a specific tool to assess PCS; however, participants were asked questions about various health conditions and symptoms consistent with several, but not all, questions included in the RPCQ.^43^ This survey guided our operationalizing of individual symptoms based on the following: Participants were asked how often, in the last four weeks, they were bothered by (1) fatigue, (2) restlessness, (3) sleep disturbances, (4) poor concentration, and (5) irritability or being easily angered on a Likert scale with possible answers “Not at all,” “Several days,” or “More than half the days.” For the analysis we collapsed “Several days,” or “More than half the days,” versus “Not at all.”

Participants were also asked the frequency, in the previous two weeks, that they had been bothered by: (1) depression, (2) sleep disturbances, (3) fatigue, (4) poor concentration, and (5) slowed thinking on a Likert scale of “Not at all,” versus “Several days,” “More than half the days,” and “Nearly every day,” which were combined for the analysis.

The questions, “Has your doctor (in the last 3 years), told you that you have any of the following conditions? (yes/no)” and “Have you had persistent or recurring problems with the following? (yes/no)” captured depression, headaches, memory loss or impairment, confusion, sleep disturbances, fatigue, and forgetfulness. An answer of “yes” to any of these questions was defined as having that symptom. If the participant answered “no” to similar symptoms in one question stem, but yes in another, they were categorized as having that PCS (i.e., if they said they were not bothered by depression, but said a doctor had diagnosed them with depression, this was categorized as “yes” for depression).

For the analysis, we created the dependent variable as the total count of PCS by summing up all the PCS for which participants answered “yes.” Nine total PCS were identified and defined for this study.

Total lifetime TBI was assessed through self-report, with participants being asked, “Have you ever had an injury, such as from a fall, blow to the head, blast exposure, motor vehicle crash, sports, or any other cause that resulted in any of the following: (a) Being dazed or confused right after the injury?; (b) Being confused or not thinking clearly right after the injury?; (c) Not remembering the actual injury right after it happened?; (d) Not remembering things that happened right after the injury?; and/or (e) Losing consciousness or being knocked out?” Participants were categorized as ever having a TBI if they answered “yes” to any of these questions.

Those who experienced a TBI were then asked the number of times they experienced this before and during military service. For our TBI variable, those who reported never having a TBI were categorized as none, while the summation of those who did was categorized as one, two, three, and four or more.

Covariates included years of age, sex (male/female), and race/ethnicity (White, Black, Hispanic, Asian/Pacific Islander, and American Indian/Other), military rank (enlisted/officer), military occupational category (MOC— Administration & Executives, Infantry/Tactical Operations, Electronics Repair & Engineering, Communications & Intelligence, Health Care, Technical & Professional, Craftworkers & Supply, Other/Not Specified), military status (active duty, Reserve, National Guard), and military branch (Army, Air Force, Coast Guard, Marine Corps, Navy).

We also included post-traumatic stress disorder (PTSD), which was assessed using the validated Posttraumatic Stress Disorder Civilian Checklist (PCL-C). The checklist measures PTSD symptoms on a scale of 17 to 85 and requires a summed score of at least 50 for an individual to be categorized as experiencing PTSD. We categorized PTSD as yes/no based on the cutoff score of at least 50. Participants were also asked about negative combat experiences that included eight questions about experiences such as seeing dead bodies or knowing someone killed. The total experiences were summed and categorized as 0, 1, 2, 3 or 4+.

Time in service for each panel was calculated by estimating the midpoint for time each panel likely would have entered the military based on enrollment criteria for the MilCo Study up until the survey was released to participants in 2014.

### Statistical analyses

Frequencies and means were used to describe the sample population demographics and occupational characteristics. The frequencies of service-related lifetime TBIs were calculated for each participant as well as the overall and individual PCS. Bivariate analyses were conducted for the total number of TBIs, as well as the number of negative combat experiences, by the number of PCS (grouped as 0, 1-2, 3-5, ≥6), with associations examined using chi-square testing.

We also examined the total lifetime TBIs by the mean and standard error of the total number of PCS using Kruskal-Wallis testing. To examine the associations between lifetime TBIs and counts of PCS, we calculated unadjusted and adjusted prevalence ratios (PRs) and 95% confidence intervals (CI) using multi-variable zero-inflated negative binomial regression models; the offset was time in service. Any variable changing the adjusted relationship between TBI and PCS by more than 10% was retained in the final model.

Further, variables were evaluated for inclusion in the model using purposeful selection, as described by Hosmer and Lemeshow.^61^ Model fit was evaluated with likelihood ratio tests and Akaike Information Criteria (AIC). All included variables improved the model fit and were statistically significant by the likelihood ratio test. The variance of PCS at each level of TBI was more than twice the mean at each level, and the alpha parameter indicated overdispersion was present, indicating Poisson count models were likely to be a poor fit of the data.

Further, 28% of participants reported zero PCS, indicating zero inflation may be present. Based on the AICs, a zero-inflated negative binomial (ZINB) model was a better fit compared with negative binomial and Poisson models. Therefore, we report findings obtained from the ZINB models used for final reporting. Statistical analyses were conducted using R^54^ (univariate, bivariate, Kruskal-Wallis testing) and Stata version 14.2^55^ (model building and analysis) statistical software programs.

## Results

Demographic details for the sample are included in Table 1. Approximately 31% of participants reported experiencing one or more TBI during their lifetime. The average age of the sample was 34.3 (standard deviation [SD] = 5.5) years, with participants being predominately male (70.5%), White (74.6%), Enlisted (66.9%), on active duty status (67.2%), and serving in the Army (40.8%), Air Force (35.5%), Navy (14.6%), Marines (6.1%), and Coast Guard (3.0%). Participants worked in the following MOCs: “Electronic Repair and Engineering” (17.2%), “Infantry/Tactical Operations” (15.7%), “Craftworkers and Supply” (13.3%), “Health Care” (11.9%), “Communications and Intelligence” (11.5%), “Administration and Executives” (9.9%), and “Technical and Professional” (5.5%), with 15.0% in an unspecified occupation (i.e., students, patients).

**Table 1.** Sample Characteristics, 2014-2016 Millennium Cohort Survey Respondents (N = 28,263)

Demographics	*n *(%)
Total lifetime TBI	
None	19,494 (69.0)
1	3,491 (12.4)
2	2,139 (7.6)
3	1,392 (4.9)
4+	1,747 (6.2)
Age, M (SD)	34.3 (5.5)
Sex	
Female	8,332 (29.5)
Male	19,931 (70.5)
Race	
White	21,081 (74.6)
Black	2,534 (9.0)
Hispanic	2,239 (7.9)
Asian/Pacific Islander	1,664 (5.9)
American Indian/Other	745 (2.6)
Rank	
Officer	9,365 (33.1)
Enlisted	18,898 (66.9)
Military status	
Reserve	9,261 (32.8)
Active Duty	19,002 (67.2)
Military occupation	
Administration & Executives	2,812 (9.9)
Infantry/Tactical Operations	4,441 (15.7)
Elec Repair and Engineering	4,850 (17.2)
Communications & Intelligence	3,246 (11.5)
Health Care	3,360 (11.9)
Technical & Professional	1,557 (5.5)
Craftworkers & Supply	3,759 (13.3)
Other/Not Specified	4,238 (15.0)
Branch of service	
Air Force	10,040 (35.5)
Army	11,530 (40.8)
Navy	4,126 (14.6)
Marine Corps	1,720 (6.1)
Coast Guard	847 (3.0)
PTSD	
No PTSD	26,233 (92.8)
Yes PTSD	2,030 (7.2)
Negative combat experiences	
None	21,300 (75.4)
1	3,170 (11.2)
2	1,606 (5.7)
3	1,181 (4.2)
4+	1,006 (3.6)

PTSD, post-traumatic stress disorder.

A small proportion of the sample (7.2%) met the criteria for PTSD, and approximately 24.6% reported negative combat experiences. Average time in service was 11.5 years for panel 2, 9 years for panel 3, and 3.5 years for panel 4.

Overall, 72% of the sample reported having at least one PCS. Table 2 displays the proportion of service members who reported each of the nine PCS. The most reported PCS was fatigue (54.0%) followed by sleep disturbances (49.7%), irritability (42.1%), poor concentration (26.4%), feeling depressed (24.5%), poor memory (21.8%), headache (20.3%), restlessness (19.2%), and slowed thinking (8.5%).

**Table 2.** Frequency (N and %) of Respondents With Self-Reported Post-Concussive Symptoms in the 2014-2016 Millennium Cohort Survey Respondents (N = 28,263)

Symptoms	Overall
	*N *(%)
Being irritable, easily angered	11,901 (42.1)
Fatigue	15,268 (54.0)
Feeling depressed	6,919 (24.5)
Headache	5,731 (20.3)
Poor concentration	7,455 (26.4)
Poor memory	6,149 (21.8)
Sleep disturbance	14,058 (49.7)
Slowed thinking	2,412 (8.5)
Restlessness	5,436 (19.2)
At least one of the above symptoms	20,232 (71.6)

Total lifetime TBI and total negative combat experiences reported by service members by the total number of PCS are displayed in Table 3. We observed differences between number of PCS and both total lifetime TBI (*p* < 0.001) and negative combat experiences (*p* < 0.001). Those with no history of TBI had the smallest proportion of participants reporting six or more PCS (12.9%). As the number of total lifetime TBI increased, the proportion of participants reporting six or more PCS also increased, with 18.1% of those with one lifetime TBI, 25.8% of those with two lifetime TBI, and 31.8% of those with three or more lifetime TBI reporting six or more PCS. Of those reporting four or more lifetime TBI (*n* = 1747), only 1.0% reported no PCS while 41.8% reported six or more PCS.

**Table 3.** Frequency (N and %) Of Respondents With Traumatic Brain Injuries and Negative Combat Experiences by Number of Self-Reported Post-Concussive Symptoms Among 2014-2016 Millennium Cohort Survey Respondents (N = 28,263)

	Number of post-concussive symptoms					
	Total (*n*)	0	1 - 2	3 - 5	6+	^a^ *p*
Total lifetime TBI						< 0.001
None	19,494	6,503 (33.4)	5,593 (28.7)	4,888 (25.0)	2,510 (12.9)	
1	3,491	800 (23.0)	1,031 (29.5)	1,029 (29.4)	631 (18.1)	
2	2,139	372 (17.4)	538 (25.1)	678 (31.7)	551 (25.8)	
3	1,392	190 (13.7)	330 (23.7)	429 (30.8)	443 (31.8)	
4+	1,747	166 (1.0)	312 (17.9)	538 (30.8)	731 (41.8)	
Total NCE						< 0.001
None	21,300	6,665 (31.3)	5,995 (28.2)	5,523 (25.9)	3,117 (14.6)	
1	3,170	682 (21.6)	870 (27.5)	913 (28.8)	705 (22.2)	
2	1,606	343 (21.4)	412 (25.6)	493 (30.7)	358 (22.3)	
3	1,181	198 (16.8)	295 (24.9)	335 (28.4)	353 (29.9)	
4+	1,006	143 (14.2)	232 (23.1)	298 (29.6)	333 (33.1)	

TBI, traumatic brain injury; NCE, negative combat experiences; ^a^chi-square test.

The same pattern was found for negative combat experiences and PCS. As the number of negative combat experiences increased, the proportion of participants reporting higher PCS also increased, with 33.1% of those reporting four or more negative combat experiences also reporting six or more PCS.

We observed differences between PCS and lifetime TBI (*p* < 0.001) and total negative combat experiences (*p* < 0.001). As the mean number of PCS increased, mean lifetime TBI and mean negative combat experiences increased as well. The mean number of PCS reported by those with four or more TBI (4.63) was more than twice that of those reporting no lifetime TBI (2.28).

Effect estimates for the unadjusted and adjusted PRs with 95% CIs from the models are reported in Table 4. In the unadjusted and adjusted models, we observed that as the number of TBIs increased, the mean number of self-reported PCS also increased. These associations were attenuated in the adjusted model; however, these associations remained for those with one, two, three, and four or more TBI to 1.10 (95% CI: 1.06–1.15), 1.19 (95% CI: 1.14–1.25), 1.23 (95% CI: 1.17–1.30), and 1.30 times (95% CI: 1.24–1.37), respectively.

**Table 4.** Association of Traumatic Brain Injuries, Post-Traumatic Stress Disorder, and Demographics With Self-Reported Post-Concussive Symptoms in the 2014-2016 Millennium Cohort Survey Respondents (N = 28,263)

Variable	***Unadjusted PR (95% CI)***		***Adjusted PR (95% CI) Model 1***		***Adjusted PR (95% CI) Model 2***	
Total lifetime TBI						
None	1.00	Referent	1.00	Referent	1.00	Referent
1	1.12	(1.08 - 1.18)	1.18	(1.14 - 1.23)	1.10	(1.06 - 1.15)
2	1.31	(1.24 - 1.38)	1.33	(1.27 - 1.39)	1.19	(1.14 - 1.25)
3	1.46	(1.38 - 1.55)	1.42	(1.35 - 1.51)	1.23	(1.17 - 1.30)
4+	1.70	(1.61 - 1.78)	1.51	(1.44 - 1.59)	1.30	(1.24 - 1.37)
PTSD						
No PTSD	1.00	Referent	1.00	Referent	1.00	Referent
Yes PTSD	2.74	(2.63 – 2.85)	2.68	(2.57 - 2.80)	2.40	(2.30 - 2.50)
Age	0.97	(0.97 - 0.98)			0.97	(0.97 - 0.97)
Sex						
Female	1.00	Referent			1.00	Referent
Male	0.93	(0.90 - 0.96)			0.81	(0.79 - 0.84)
Race						
White	1.00	Referent			1.00	Referent
Black	1.01	(0.96 - 1.07)			0.90	(0.86 - 0.94)
Hispanic	1.18	(1.12 - 1.25)			1.02	(0.98 - 1.07)
Asian/Pacific Islander	1.01	(0.95 - 1.08)			0.98	(0.93 - 1.04)
American Indian/Other	1.17	(1.07 - 1.28)			1.13	(1.05 - 1.22)
Rank						
Officer	1.00	Referent			1.00	Referent
Enlisted	1.52	(1.47 - 1.57)			1.35	(1.31 - 1.39)
Military status						
Reserve	1.00	Referent			1.00	Referent
Active Duty	0.93	(0.90 – 0.96)			1.03	(1.00 – 1.06)
Branch of service						
Air Force	1.00	Referent			1.00	Referent
Army	1.43	(1.38 - 1.48)			1.43	(1.38 - 1.47)
Navy	1.28	(1.23 - 1.35)			1.37	(1.31 - 1.42)
Marine Corps	1.46	(1.37 - 1.56)			1.38	(1.31 - 1.46)
Coast Guard	1.16	(1.06 - 1.28)			1.21	(1.12 - 1.31)
Negative combat experiences						
None	1.00	Referent			1.00	Referent
1	1.25	(1.20 - 1.31)			1.22	(1.18 - 1.27)
2	1.25	(1.17 - 1.32)			1.17	(1.11 - 1.23)
3	1.41	(1.32 - 1.51)			1.27	(1.20 - 1.34)
4 or more	1.50	(1.40 - 1.62)			1.25	(1.17 - 1.33)
Military occupation						
Administration & Executives	1.00	Referent			1.00	Referent
Infantry/Tactical Operations	1.01	(0.95 - 1.08)			1.01	(0.95 - 1.06)
Elec Repair & Engineering	1.00	(0.94 - 1.06)			1.03	(0.98 - 1.09)
Comm. & Intelligence	1.03	(0.96 - 1.09)			1.06	(1.00 - 1.12)
Health Care	1.12	(1.05 - 1.19)			1.11	(1.05 - 1.17)
Technical & Professional	0.89	(0.82 - 0.96)			1.05	(0.98 - 1.12)
Craftworkers & Supply	1.14	(1.07 - 1.22)			1.09	(1.04 - 1.15)
Other/Not Specified	0.94	(0.88 – 1.00)			1.10	(1.04 - 1.16)

TBI, traumatic brain injury; PTSD, post-traumatic stress disorder; PR, Prevalence Ratio from multivariable zero-inflated negative binomial regression model; 95% CI:,95% Confidence Interval; Model 1 includes TBI and PTSD only; Model 2 includes all variables listed.

A test for trend treating the categorical TBI variable as continuous in the model showed a PR of 1.07 (95% CI 1.06–1.08). The PTSD functioned as confounder in the model and was associated with increased prevalence of PCS, because those with PTSD had 2.40 times (95% CI: 2.30–2.50) higher prevalence of PCS compared with those without PTSD. Being male was protective of PCS, with a reduced prevalence of 0.81 (95% CI: 0.79–0.84) of PCS reported relative to females. Only the race American Indian/Other positively associated with PCS (PR 1.13; 95% CI: 1.05 – 1.22) compared with White service members.

Enlisted service members had 1.35 times more PCS (95% CI: 1.31–1.39) compared with officers. For each branch of service, the prevalence of PCS was higher for Army (PR: 1.43, 95% CI: 1.38–1.47), Marines (PR: 1.38, 95% CI: 1.31–1.46), Navy (PR: 1.37 95% CI: 1.31–1.46), and Coast Guard (PR: 1.21, 95% CI: 1.12–1.31) compared with their Air Force counterparts.

Negative combat experiences were also associated with increased prevalence of PCS. Those with one (PR: 1.22, 95% CI: 1.18–1.27), two (PR: 1.17, 95% CI: 1.11–1.23), three (PR: 1.27, 95% CI: 1.20–1.34), or four or more (PR: 1.25, 95% CI: 1.17–1.33) negative combat experiences had higher prevalence of PCS compared with those with no negative combat experiences.

Only a few military occupational categories had increased prevalence of PCS compared with those in the “Administration & Executives” category: “Health Care (PR: 1.11, 95% CI: 1.05–1.17),” “Craftworkers & Supply (PR: 1.09, 95% CI: 1.04–1.15),” and “Other/Not Specified (OR: 1.10, 95% CI: 1.04–1.16).”

Associations between number of TBI and PCS remained with similar effect measures for the model including only PTSD as a confounder. The prevalence of PCS was 2.68 (95% CI: 2.57–2.80) times higher in those with PTSD compared with those without PTSD. Reporting four or more lifetime TBI was associated with a 51% (PR 1.51, 95% CI: 1.44–1.59) increased prevalence of PCS compared with those reporting no lifetime TBI.

## Discussion

This study is the first to examine the relationship between total count of lifetime TBI, including TBI experienced outside of military service, and PCS among U.S. active duty military service members. In this study, 31% of service members reported experiencing at least one TBI and 28% reported at least one PCS. Service members reporting repeated TBI had higher prevalence of PCS than their peers who reported none or one TBI. We also observed that as the number of TBI increased, the number of PCS increased.

These results support previous research that has found those reporting TBI to be significantly more likely to report PCS than those with no history of TBI in military populations.^16,44^ A cross-sectional analysis of 398 veterans from three VA Polytrauma Network sites from August 1, 2010, to September 30, 2011 recruited veterans who had not received TBI treatment in the preceding 30 days and examined their TBI status and presence and severity of PCS. ^16^

Veterans testing negative for TBI reported experiencing an average of 9.4 symptoms overall while those who tested positive reported an average of 16.7 symptoms. Those with TBI were 30% more likely to report symptoms than those who did not have TBI. While they did not explore the number of TBI, The 2015 study by Dretch and associates^44^ did, grouping service members into TBI categories zero, one, two, and three or more. They found that concussion history predicted PCS but did not find differences between the TBI groups compared with the no TBI group until total concussions exceeded three.

Beyond military populations, these results are similar to findings in the sports literature examining the association between deleterious neurological symptoms and number of concussions, primarily among amateur and professional athletes of contact sports.^18,20,3^ When followed over nine years, retired National Football League players were at a significantly higher risk of depression as the number of self-reported concussions increased. ^20^ Players reporting 10 or more concussions had 5.8 times the risk of depression compared with players reporting no concussions. ^20^

When using age of first exposure (AFE) to football as a proxy measure for head injuries, the odds of depression were three times higher in those whose AFE was before the age of 12 versus those whose AFE was 12 or older. ^62^

A 2017 cross-sectional study of 576 professional contact sports athletes explored the relationship between concussions and symptoms of distress, anxiety, depression, sleep disturbances, and substance abuse, which they referred to as common mental disorders. Those in the six or more concussions group were at significantly increased odds of common mental disorders, sleep disturbance, and adverse alcohol use when compared with the group that reported no concussions. ^18^

A TBI is often found to be comorbid with PTSD because the TBI results from a traumatic experience (e.g., an improvised explosive device blast, combat related injury).^63,64^ Our study found increased PCS as total lifetime TBI increased, even after adjusting for PTSD. Further, these findings suggest a potential dose-response relationship between number of TBI and number of PCS experienced, even after accounting for PTSD. However, given the cross-sectional nature of this study, this relationship should be further analyzed in a more robust, longitudinal design.

Some PCS overlap with those of PTSD, depending on how PCS have been defined, yet some studies do not adjust for this condition.^1,2,44,52,65,66^ A 2015 cross-sectional analysis of 51,201 veterans found those with a history of any mTBI reported 30% more PCS when compared with their peers with no history of TBI.^16^ PTSD was also associated with a 14% increase in PCS compared with those without PTSD.

A smaller cross-sectional study (2010) of service members returning from Iraq found the prevalence of any PCS was four times higher in those who screened positive for mTBI and negative for PTSD when compared with those who screened negative for mTBI and negative for PTSD. The prevalence of PCS, however, was 6.3 times higher in those who screened positive for both TBI and PTSD compared with those who screened negative for TBI and PTSD.^48^

When considering TBI, Drestch and colleagues^44^ (*n* = 469) found a significant increase in PCS as TBI increased from zero to one, one to two, two to three, and three or more. A retrospective analysis of 22,203 Special Operations soldiers between November 1, 2009, to December 31, 2011, examined the relationship between TBI and residual symptoms, PTSD, and neurocognitive performance.^52^ TBI was categorized in three ways by mechanism as none, blunt only, blast only, or a combination of both. Those with a history of diagnosed blast and combination TBI reported more post-concussive symptoms and were more likely to screen positive for PTSD than those reporting only blunt TBI or no TBI at all.

When considering multiple TBI, in any combination or singularly, the researchers grouped TBI as zero, one, two, and three or more. They found a significant dose-response relationship, wherein as history of TBI increased, PCS and odds of PTSD also increased (*p* trend = 0.001). While this study provided some evidence of a dose-response relationship between TBI and PCS, the population studied was highly specialized, making it difficult to compare and generalize across all military branches and occupations, which may not have similar confounding exposure.

This study can be generalized to the military community at large because there were only minor differences between the study sample's demographics and those of the U.S. military.^67^ The sample was older, with the average age of the study sample around 34 years while the DOD reports an average age of 28.2 years.^67^ Except for age, the remaining demographics of general U.S. military personnel were similar to those of the 2018 survey, with service members being mostly male (71%), White (75%), enlisted (67%), and serving in the Army (36%).^67^

Our findings need to be considered in view of some methodological limitations. First, the cross-sectional design and use of existing survey data limit our ability to establish any causality or directionality of the associations between TBI and PCS. Given this study used existing survey data, clinical imaging and observations were unavailable, which may have impacted results by including biological and physiological measures associated with neurobehavioral dysfunction.

Second, the measurement of TBI and PCS were based on self-reports and, therefore, potentially subject to recall bias. This may especially be the case for those participants who may have memory issues from TBI. Third, the PCS were not captured with a validated PCS instrument, but instead derived from individual health questions from the survey. This could have biased results away from the null as PCS may be non-specific and could have been attributed to other conditions. In addition, some symptoms, such as fatigue and those related to sleep, may be from environmental and occupational conditions inherent in military service and training and may not be directed related to any specific health condition.

Fourth, there is also the potential for selection bias. Service members and veterans who continue to answer Millennium Cohort Surveys every three to four years may be those service members who have health problems that they hope the surveys will help answer. As such, they may self-select into continued surveys as the prospective study continues. It is also possible that the extremely ill or disabled cohort members do not continue to respond to surveys because of their condition.

A strength of this study is the consideration of lifetime TBI exposure rather than a binary outcome. This allowed us to visualize and test associations of repeated TBI with PCS, including associations between PCS as the number of TBI increased. Further, we considered lifetime TBI exposures, not just those occurring within military service.

Another strength was the large and diverse cross-section of military service members across all branches, to include the Coast Guard. We not only included Coast Guard service members, but we also calculated prevalence estimates in comparison with the other branches of military service, providing data to an existing gap in the scientific literature. The increased odds of PCS for all branches compared with the Air Force deserves further investigation although this finding may be related to a higher proportion of service members in other branches reporting higher TBI, thereby increasing the risk of PCS in these other branches of service.^53,54^

Given the associations found between repeated TBI and PCS, further exploration of longitudinal data is warranted in which a temporal association between TBI and PCS can be established. It will also be imperative to use a gold-standard measurement for capturing PCS, such as the NSI or RPCQ.

Finally, we believe our inclusion of military occupation as a variable is a strength of our study because it includes another potential risk factor for TBI. An unexpected finding related to occupation was that of an increased risk of PCS among “Health Care” professions compared with “Administration & Executives.” While “Health Care” professions may be thought to be less risky, in the military, this would include occupations within Special Operations Forces, such as Combat Medics, which have reported higher neurobehavioral symptoms than conventional forces.^68^

Another potential hypothesis for this finding is that “Health Care” occupations may be at higher risk of negative combat experiences in seeing fellow service members they care for in extreme peril, or worse, after injury in combat. This may increase the risk of post-traumatic stress among this occupational group, which would overlap with PCS. This finding deserves further investigation.

## Conclusion

These results suggest an elevated prevalence of PCS as the number of TBI increased. This highlights the need for robust, longitudinal studies that can establish a temporal relationship between repetitive TBI and incidence of PCS. Continued development of personal protective equipment, improved technology in transportation, and logistics to reduce the risk of TBI is also necessary. These results may be most relevant to the Defense Health Agency (DHA) and DoD as they further the Warfighter Brain Health Initiative, especially when considering occupation as a potential risk factor for negative brain health outcomes.

Finally, the increased risk of PCS with each repeated TBI should be further investigated by these entities when considering long-term brain health, because this may mean special considerations and interventions for individuals with multiple TBI.

## Data Process

A data sharing agreement must be executed with the original data owner (The Naval Health Research Center, San Diego) to obtain the data with appropriate permissions. The code is available in R format from the primary author.

Disclaimer: I am a military service member or employee of the U.S. Government. This work was prepared as part of my official duties. Title 17, U.S.C. §105 provides that copyright protection under this title is not available for any work of the U.S. Government. Title 17, U.S.C. §101 defines a U.S. Government work as work prepared by a military service member or employee of the U.S. Government as part of that person's official duties. Report No. 21-71 was supported by the National Institute for Occupational Safety and Health (NIOSH) Southwest Center for Occupational and Environmental Health (SWCOEH) Education and Research Center under work unit no. 60002/ Grant No. 5T42OH008421. The views expressed in this article are those of the authors and do not necessarily reflect the official policy or position of the Henry M. Jackson Foundation for the Advancement of Military Medicine, Inc.; Department of the Navy, Department of Defense, nor the U.S. Government. The study protocol was approved by the Naval Health Research Center Institutional Review Board in compliance with all applicable Federal regulations governing the protection of human subjects. Research data were derived from an approved Naval Health Research Center Institutional Review Board protocol number NHRC.2000.0007. The opinions and assertions expressed herein are those of the author(s) and do not necessarily reflect the official policy or position of the Uniformed Services University or the Department of Defense.
